# Supplementary material for: The impact of the management strategies for patients with subclinical hypothyroidism on long-term clinical outcomes: An umbrella review
Source: PLoS One. 2022 May 19;17(5):e0268070. doi: 10.1371/journal.pone.0268070 (PMC9119548; doi:10.1371/journal.pone.0268070)
Supplement: S1 Appendix — (PDF) [file pone.0268070.s001.pdf]

Additional File 1. MEDLINE Search Strategy

Database: Ovid MEDLINE(R) and Epub Ahead of Print, In-Process & Other Non-Indexed Citations, Daily and Versions(R) <1946 to February 17, 2021>
Search Strategy:
----

1 exp Hypothyroidism/ (33056)
2 exp Thyroid Diseases/ (149791)
3 hypothyroid*.tw. (35912)
4 (thyroid? adj3 deficien*).tw. (1536)
5 (thyroid? adj3 insufficien*).tw. (488)
6 (thyroid? adj3 failure?).tw. (549)
7 (thyroid? adj3 low adj3 product*).tw. (7)
8 (thyroid? adj3 under adj3 product*).tw. (4)
9 (thyroid? adj3 underactiv*).tw. (27)
10 (thyroid? adj3 hypofunction).tw. (265)
11 (thyroid? adj3 d?sfunction).tw. (5642)
12 or/1-11 (162773)
13 (mild* or sub-clinic* or subclinic*).tw. (453666)
14 12 and 13 (8752)
15 (tsh adj3 (elevat* or increas* or high*)).tw. (6791)
16 (SHT or SCH).tw. (8664)
17 14 or 15 or 16 (22418)
18 exp Hormone Replacement Therapy/ (25185)
19 (thyroid? adj3 (therap* or treat*)).tw. (12185)
20 exp Thyroxine/ (48783)
21 (thyroxin* or levothyrox* or levo-thyrox* or l-thyrox* or L-T4 or LT4).tw. (34600)
22 or/18-21 (94909)
23 (follow?up or monitor* or observ* or surveil*).tw. (4488288)
24 ((no or lack*) adj2 (treatment* or therap* or intervention*)).tw. (137605)
25 (untreated or ('not' adj2 treat*)).tw. (264180)
26 or/23-25 (4777064)
27 exp Cardiovascular diseases/ or exp Heart diseases/ or exp Myocardial ischemia/ or exp Vascular diseases/ or exp Arteriosclerosis/ (2435564)
28 Carotid Intima Media Thickness/ or Intima-Media Thickness, Carotid/ or Atherosclerosis/ or Atheroscleroses/ or Atherogenesis/ (41075)

29 ((cardiovasc* or vasc* or cardio* or cardia* or heart* or coronary* or myocard* or pericard* or isch$em*) adj2 (disease? or event? or arrest? or fail* or mortality)).tw. (745362)
30 (lipid$ or cholesterol or TC or triglyceride$ or LDL or HDL).tw. (756725)
31 (myocardi* adj (infarct* or revascular* or re-vascular* or isch$emi*)).tw. (225198)
32 (heart attack* or angina).tw. (58869)
33 (morbid* adj5 (cardio* or cardia* or heart* or coronary* or myocard* or pericard* or isch$em*)).tw. (25873)
34 peripheral arter* disease*.tw. (14503)
35 (emboli* or arrhythmi* or thrombo* or atrial fibrillat* or atrial flutter* or tachycardi* or endocardi* or (sick adj sinus)).tw. (690392)
36 (flow-mediated vasodilat* or flow-mediated dilat* or endothelial-dependent vasodilat* or endothelial-dependent dilat* or endothelial function$ or carotid intima-media thickness or intima-media thickness or carotid-wall thickness or carotid atherosclerosis or C-IMT).tw. (35362)
37 (isch$emi* adj2 (vascular or heart)).tw. (34721)
38 or/27-37 (3563857)
39 exp Quality of Life/ (204526)
40 quality of life.tw. (295840)
41 (QoL or HRQoL).tw. (56831)
42 or/39-41 (359236)
43 exp Stroke/ (140280)
44 exp Ischemic Attack, Transient/ (20638)
45 (stroke$ or apoplexy).tw. (263191)
46 ((cerebrovasc* or cerebral vascular or brain) adj2 (disease? or event? or arrest? or fail* or mortality or accident* or death*)).tw. (62563)
47 ((brain* or cerebral or lacunar) adj2 infarct*).tw. (27518)
48 (isch$emi* adj2 (transient or attack* or cerebral or brain)).tw. (57232)
49 or/43-48 (387778)
50 exp Frailty/ (3592)
51 frail*.tw. (24209)
52 exp Fractures, Bone/ or fracture$.tw. (310642)
53 (50 or 51) and 52 (1293)
54 (mortality or death*).tw. (1479781)
55 Meta-Analysis as Topic/ (19028)
56 meta analy$.tw. (194305)
57 metaanaly$.tw. (2211)
58 Meta-Analysis/ (126788)

59 (systematic adj (review$1 or overview$1)).tw. (197114)

60 exp Review Literature as Topic/ (15372)

61 or/55-60 (335876)

62 cochrane.ab. (94602)

63 embase.ab. (105156)

64 (psychlit or psyclit).ab. (915)

65 (psychinfo or psycinfo).ab. (40574)

66 (cinahl or cinhal).ab. (32163)

67 science citation index.ab. (3252)

68 bids.ab. (563)

69 cancerlit.ab. (633)

70 or/62-69 (170652)

71 reference list$.ab. (19037)

72 bibliograph$.ab. (19191)

73 hand-search$.ab. (7333)

74 relevant journals.ab. (1216)

75 manual search$.ab. (4784)

76 or/71-75 (46231)

77 selection criteria.ab. (31649)

78 data extraction.ab. (23699)

79 77 or 78 (52968)

80 Review/ (2767148)

81 79 and 80 (29899)

82 Comment/ (894822)

83 Letter/ (1124632)

84 Editorial/ (558685)

85 animal/ (6756006)

86 human/ (19036693)

87 85 not (85 and 86) (4755937)

88 or/82-84,87 (6623165)

89 61 or 70 or 76 or 81 (402770)

90 89 not 88 (382792)

91 17 and (22 or 26) and (38 or 42 or 49 or 53 or 54) (2382)

92 90 and 91 (96)
