# Supplementary material for: The impact of the management strategies for patients with subclinical hypothyroidism on long-term clinical outcomes: An umbrella review
Source: PLoS One. 2022 May 19;17(5):e0268070. doi: 10.1371/journal.pone.0268070 (PMC9119548; doi:10.1371/journal.pone.0268070)
Supplement: S3 Appendix — (PDF) [file pone.0268070.s003.pdf]

List of papers excluded after full-text screening

Not a systematic review or meta-analysis (n = 23)

1. Leng O, Razvi S. Treatment of subclinical hypothyroidism: assessing when treatment is likely to be beneficial. Expert Review of Endocrinology & Metabolism. 2021 Mar 4;16(2):73-86.
2. Panday P, Franchini AP, Iskander B, Anwer F, Oliveri F, Kakargias F, Hamid P. Subclinical hypothyroidism in geriatric population and its association with heart failure. Cureus. 2021 Apr 5;13(4).
3. Apostu D, Lucaciu O, Oltean-Dan D, Mureșan AD, Moisescu-Pop C, Maxim A, Benea H. The influence of thyroid pathology on osteoporosis and fracture risk: A review. Diagnostics. 2020 Mar;10(3):149.
4. Biondi B, Palmieri EA, Lombardi G, Fazio S. Effects of subclinical thyroid dysfunction on the heart. Annals of internal medicine. 2002 Dec 3;137(11):904-14.
5. Delitala AP, Scuteri A, Maioli M, Mangatia P, Vilardi L, Erre GL. Subclinical hypothyroidism and cardiovascular risk factors. Minerva Medica. 2019 Nov 11;110(6):530-45.
6. Chrysant SG. The current debate over treatment of subclinical hypothyroidism to prevent cardiovascular complications. International Journal of Clinical Practice. 2020 Jul;74(7):e13499.
7. Tognini S, Pasqualetti G, Calsolaro V, Polini A, Monzani F. Cognitive function and quality of life in mild thyroid hormone deficiency. Recent patents on endocrine, metabolic & immune drug discovery. 2014 May 1;8(2):124-34.
8. Thvilum M, Brandt F, Brix TH, Hegedüs L. A review of the evidence for and against increased mortality in hypothyroidism. Nature Reviews Endocrinology. 2012 Jul;8(7):417-24.
9. Triggiani V, Angelo Giagulli V, De Pergola G, Licchelli B, Guastamacchia E, Iacoviello M. Mechanisms explaining the influence of subclinical hypothyroidism on the onset and progression of chronic heart failure. Endocrine, Metabolic & Immune Disorders-Drug Targets (Formerly Current Drug Targets-Immune, Endocrine & Metabolic Disorders). 2016 Mar 1;16(1):2-7.
10. Surks MI, Ortiz E, Daniels GH, Sawin CT, Col NF, Cobin RH, Franklyn JA, Hershman JM, Burman KD, Denke MA, Gorman C. Subclinical thyroid disease: scientific review and guidelines for diagnosis and management. Jama. 2004 Jan 14;291(2):228-38.
11. Helfand M. Screening for subclinical thyroid dysfunction in nonpregnant adults: a summary of the evidence for the US Preventive Services Task Force. Annals of internal medicine. 2004 Jan 20;140(2):128-41.
12. Sgarbi JA, Teixeira PF, Maciel LM, Mazeto GM, Vaisman M, Montenegro Junior RM, Ward LS. The Brazilian consensus for the clinical approach and treatment of subclinical hypothyroidism in adults: recommendations of the thyroid Department of the Brazilian Society of Endocrinology and Metabolism. Arquivos Brasileiros de Endocrinologia & Metabologia. 2013;57:166-83.
13. Allan GM, Morros MP, Young J. Subclinical hypothyroidism and TSH screening. Canadian Family Physician. 2020 Mar 1;66(3):188-188.

14. Mariotti S, Cambuli VM. Cardiovascular risk in elderly hypothyroid patients. Thyroid. 2007 Nov 1;17(11):1067-73.
15. Marrakchi S, Kanoun F, Idriss S, Kammoun I, Kachboura S. Arrhythmia and thyroid dysfunction. Herz. 2015 Apr;40(2):101-9.
16. Hennessey JV, Espaillat R. Reversible morbidity markers in subclinical hypothyroidism. Postgraduate Medicine. 2015 Jan 2;127(1):78-91.
17. Velkeniers B, Van MA, Unuane D, Haentjens P. A critical synopsis of meta-analysis in the field of subclinical thyroid disease. In Endocrine Abstracts 2010 Apr 1 (Vol. 22). Bioscientifica.
18. Johnson BE. Thyroid hormone therapy does not improve QoL or symptoms in subclinical hypothyroidism. Annals of Internal Medicine. 2019 Feb;170(4):JC17.
19. Lee, J. H. Early detection and management of SCH is important. Journal of Family Practice. 2006; 55 (6):543
20. Lucas-Martín AM. Hipotiroidismo subclínico: tratar o no tartar. Medicina Clínica. 2004; 122(5):182-183. doi:https://doi.org/10.1016/S0025-7753(04)74187-1.
21. Rodondi N, Maisonneuve P, Razvi S, Elzen WD, Gussekloo J, Iervasi G, Asvold BO, Imaizumi M, Vanderpump M, Westendorp RG, Franklyn JA. Subclinical Hypothyroidism and the Risk of Coronary Heart Disease and Mortality: An Individual Participant Data Analysis from Nine Prospective Cohort Studies. Journal of General Internal Medicine. 2010 Jun 30;25:394-5.
22. Gencer B, Collet TH, Virgini V, Auer R, Rodondi N. Subclinical thyroid dysfunction and cardiovascular outcomes among prospective cohort studies. Endocrine, Metabolic & Immune Disorders-Drug Targets (Formerly Current Drug Targets-Immune, Endocrine & Metabolic Disorders). 2013 Mar 1;13(1):4-12.
23. Rugge B, Balshem H, Sehgal R, et al. Screening and Treatment of Subclinical Hypothyroidism or Hyperthyroidism. Agency for Healthcare Research and Quality (US), Rockville (MD); 2011. PMID: 22299183.

No reported management (treated or not treated) (n = 7)

1. Haentjens P, Van Meerhaeghe A, Poppe K, Velkeniers B. Subclinical thyroid dysfunction and mortality: an estimate of relative and absolute excess all-cause mortality based on time-to-event data from cohort studies. European Journal of Endocrinology. 2008 Sep 1;159(3):329-41.
2. Zhu H, Zhang J, Wang J, Zhao X, Gu M. Association of subclinical thyroid dysfunction with bone mineral density and fracture: a meta-analysis of prospective cohort studies. Endocrine. 2020 Mar;67(3):685-98.
3. Tsai TY, Tu YK, Munir KM, Lin SM, Chang RH, Kao SL, Loh CH, Peng CC, Huang HK. Association of hypothyroidism and mortality in the elderly population: a systematic review and meta-analysis. The Journal of Clinical Endocrinology & Metabolism. 2020 Jun 1;105(6):2068-80.
4. Larsson SC, Allara E, Mason AM, Michaëlsson K, Burgess S. Thyroid function and dysfunction in relation to 16 cardiovascular diseases: a Mendelian randomization study. Circulation: Genomic and Precision Medicine. 2019 Mar;12(3):e002468.

5. Ning Y, Cheng YJ, Liu LJ, Sara JD, Cao ZY, Zheng WP, Zhang TS, Han HJ, Yang ZY, Zhang Y, Wang FL. What is the association of hypothyroidism with risks of cardiovascular events and mortality? A meta-analysis of 55 cohort studies involving 1,898,314 participants. BMC medicine. 2017 Dec;15(1):1-5.
6. Moon S, Kim MJ, Yu JM, Yoo HJ, Park YJ. Subclinical hypothyroidism and the risk of cardiovascular disease and all-cause mortality: a meta-analysis of prospective cohort studies. Thyroid. 2018 Sep 1;28(9):1101-10.
7. Brenta G, Vaisman M, Sgarbi JA, Bergoglio LM, Andrada NC, Bravo PP, Orlandi AM, Graf H. Clinical practice guidelines for the management of hypothyroidism. Arquivos Brasileiros de Endocrinologia & Metabologia. 2013;57:265-91.

No primary outcome (n = 5)

1. He W, Li S, Zhang JA, Zhang J, Mu K, Li XM. Effect of levothyroxine on blood pressure in patients with subclinical hypothyroidism: a systematic review and meta-analysis. Frontiers in endocrinology. 2018:454.
2. Gómez-Izquierdo J, Filion KB, Boivin JF, Azoulay L, Pollak M, Yu OH. Subclinical hypothyroidism and the risk of cancer incidence and cancer mortality: a systematic review. BMC endocrine disorders. 2020 Dec;20(1):1-0.
3. Gibbons VA. The Epidemiology and Management of Hypothyroidism in General Practice 2011 (Doctoral dissertation, University of Auckland).
4. Burgos N, Toloza FJ, Singh Ospina NM, Brito JP, Salloum RG, Hassett LC, Maraka S. Clinical outcomes after discontinuation of thyroid hormone replacement: a systematic review and meta-analysis. Thyroid. 2021 May 1;31(5):740-51.
5. Segna D, Bauer DC, Feller M, Schneider C, Fink HA, Aubert CE, Collet TH, da Costa BR, Fischer K, Peeters RP, Cappola AR. Association between subclinical thyroid dysfunction and change in bone mineral density in prospective cohorts. Journal of internal medicine. 2018 Jan;283(1):56-72.

Reported markers/midpoints rather than events (n = 3)

1. Chen Y, Tai HY. Levothyroxine in the treatment of overt or subclinical hypothyroidism: a systematic review and meta-analysis. Endocrine Journal. 2020:EJ19-0583.
2. Faber J, Galløe AM. Changes in bone mass during prolonged subclinical hyperthyroidism due to L-thyroxine treatment: a meta-analysis. European journal of Endocrinology. 1994 Apr 1;130(4):350-6.
3. Peng L, GU MJ. Influence of thyroxine treatment on serum lipid levels in patients with subclinical hypothyroidism: a Meta-analysis. Academic Journal of Second Military Medical University. 1985.

Wrong population (n = 4)

1. Gawlik A, Such K, Dejner A, Zachurzok A, Antosz A, Malecka-Tendera E. Subclinical hypothyroidism in children and adolescents: is it clinically relevant?. International Journal of Endocrinology. 2015 Mar 29;2015.
2. Monzani A, Prodam F, Rapa A, Moia S, Agarla V, Bellone S, Bona G. Endocrine disorders in childhood and adolescence. Natural history of subclinical hypothyroidism in children and adolescents and potential effects of replacement therapy: a review. European Journal of Endocrinology. 2012 Dec 10;168(1):R1-1.
3. Salerno M, Improda N, Capalbo D. Management of endocrine disease subclinical hypothyroidism in children. European Journal of Endocrinology. 2020 Aug 1;183(2):R13-28.
4. Bona G, Prodam F, Monzani A. Subclinical hypothyroidism in children: natural history and when to treat. Journal of clinical research in pediatric endocrinology. 2013 Mar;5(Suppl 1):23.

Not subclinical hypothyroidism (n = 3)

1. Chaker L, Baumgartner C, Den Elzen WP, Collet TH, Ikram MA, Blum MR, Dehghan A, Drechsler C, Luben RN, Portegies ML, Iervasi G. Thyroid function within the reference range and the risk of stroke: an individual participant data analysis. The Journal of Clinical Endocrinology & Metabolism. 2016 Nov 1;101(11):4270-82.
2. Yang LB, Jiang DQ, Qi WB, Zhang T, Feng YL, Gao L, Zhao J. Subclinical hyperthyroidism and the risk of cardiovascular events and all-cause mortality: an updated meta-analysis of cohort studies. European Journal of Endocrinology. 2012 Jul 1;167(1):75.
3. Wiersinga WM. Should we treat mild subclinical/mild hyperthyroidism? Yes. European Journal of Internal Medicine. 2011 Aug 1;22(4):324-9.

Full review published in a separate paper (n = 2)

1. Collet TH, Aujesky D, Vittinghoff E, Bauer D, Gussekloo J, Cappola AR, den Elzen WP, Sgarbi J, Cornuz J, Bremner AP, Maciel RM. Auto-immunity, subclinical hypothyroidism and the risk of coronary heart disease and mortality. Journal of General Internal Medicine. 2012 Jul 31;27:S130-S130.
2. Chaker, L.; Baumgartner, C.; Den Elzen, W. J. P.; Ikram, M. A.; Blum, M. R.; Bakker, S. J. L.; Dehghan, A.; Drechsler, C.; Luben, R. N.; Hofman, A.; Portegies, M. L. P.; Medici, M.; Iervasi, G.; Collet, T. H.; Brenmer, A.; Wanner, C.; Iacoviello, M.; Dullaart, R. P.; Sgarbi, J. A.; Ceresini, G.; Westendorp, R. G.; Jukema, J. W.; Imaizumi, M.; Franklyn, J. A.; Bauer, D. C.; Cappola, A. R.; Walsh, J. P.; Razvi, S.; Khaw, K. T.; Volzke, H.; Franco, O. H.; Gussekloo, J.; Rodondi, N.; Peeters, R. P. Subclinical hypothyroidism and the risk of non-fatal and fatal stroke: An individual participant analysis. European Thyroid Journal. 2014 August 3; 96 doi: http://dx.doi.org/10.1159/000365244

Full data unavailable (n = 2)

1. Du Puy RS, Poortvliet RK, Mooijaart SP, Den Elzen WP, Jagger C, Pearce SH, Arai Y, Hirose N, Teh R, Menzies O, Rolleston A. Outcomes of thyroid dysfunction in people aged eighty years and older: an individual patient data meta-analysis of four prospective studies (Towards Understanding Longitudinal International Older People Studies Consortium). Thyroid. 2021 Apr 1;31(4):552-62.
2. Ochs N, Auer R, Bauer DC, Nanchen D, Gussekloo J, Cornuz J, Rodondi N. Meta-analysis: subclinical thyroid dysfunction and the risk for coronary heart disease and mortality. Annals of internal medicine. 2008 Jun 3;148(11):832-45.

Reviews were updated (n = 2)

1. Chaker L, Baumgartner C, Ikram MA, Dehghan A, Medici M, Visser WE, Hofman A, Rodondi N, Peeters RP, Franco OH. Subclinical thyroid dysfunction and the risk of stroke: a systematic review and meta-analysis. European journal of epidemiology. 2014 Nov;29(11):791-800.
2. Helfand M, Redfern CC. Screening for thyroid disease: an update. Annals of internal medicine. 1998 Jul 15;129(2):144-58.
